# Influence of Dendritic Cells on B-Cell Responses during HIV Infection

**DOI:** 10.1155/2012/592187

**Published:** 2012-02-22

**Authors:** Johanne Poudrier, Josiane Chagnon-Choquet, Michel Roger

**Affiliations:** ^1^Laboratoire d'immunogénétique, Centre de Recherche du Centre Hospitalier de l'Université de Montréal (CHUM), Hôpital Notre-Dame, 2099 Alexandre De Sève, Montréal, QC, Canada H2L 2W5; ^2^Département de Microbiologie et Immunologie de l'Université de Montréal, C.P. 6128 Succ Centre-Ville, Montréal, QC, Canada H3C 3J7

## Abstract

Dendritic cells (DCs) modulate B-cell differentiation, activation, and survival mainly through production of growth factors such as B lymphocyte stimulator (BLyS/BAFF). DC populations have been reported to be affected in number, phenotype and function during HIV infection and such alterations may contribute to the dysregulation of the B-cell compartment. Herein, we reflect on the potential impact of DC on the pathogenesis of HIV-related B cell disorders, and how DC status may modulate the outcome of mucosal B cell responses against HIV, which are pivotal to the control of disease. A concept that could be extrapolated to the overall outcome of HIV disease, whereby control versus progression may reside in the host's capacity to maintain DC homeostasis at mucosal sites, where DC populations present an inherent capacity of modulating the balance between tolerance and protection, and are amongst the earliest cell types to be exposed to the virus.

## 1. Introduction

Based on the study of natural correlates of protection against HIV infection, the overall outcome of disease may depend on the host's capacity to control the extent of inflammation and preserve systemic integrity by constraining immune activity to mucosal tissues, where viral exposure occurs. There, DCs are one of the earliest cell types to be exposed to the virus and present an inherent capacity to orchestrate a homeostatic balance between tolerance and protection [1–4]. It is likely that the incapacity to keep a balance in these homeostatic processes will promote inflammation and lead to disease progression [5]. In contrast, the capacity to maintain immune homeostasis at mucosal sites will probably allow for better control of HIV-infection. The general effects of HIV-infection and disease on DC populations have been recently reviewed [1–4] and are beyond the scope of this work. This perspective review will focus on the potential impact of DCs on HIV-related B-cell disorders and responses.

Although the vast majority of HIV-infected individuals can now achieve and maintain viral suppression with modern antiretroviral therapy (ART), their life expectancy remains much shorter than the general population and they continue to be at much higher risk for non-AIDS-associated diseases commonly associated with aging. B lymphocyte dysregulations are often observed during HIV infection (reviewed in [6]), contributing to abnormal levels of immune activation and inflammation that may drive these clinical events. Given that the requirements of B-cell populations differ according to their status and stage of differentiation, they are likely to be affected differentially by the HIV context, a process reflected by events such as polyclonal activation, breakage of tolerance, altered subpopulation dynamics, exhaustion, and loss of the capacity to generate and maintain memory. All of which contribute to a global impairment of the humoral immune compartment, leading to deficiency in the generation of efficient anti-HIV responses.

Although the mechanisms involved in the triggering and progression of HIV-related B-cell disorders remain largely unknown, it has been suggested that they result from high viremia and an impaired CD4^+^ T cell compartment [6]. However, ART does not appears to fully restore the B-cell compartment since autoimmune manifestations and malignancies are still detected despite recovery of CD4^+^ T cell counts and suppression of viral replication by ART. In fact, the B-cell disease seems to progress and differ in subtype depending on the level of CD4^+^ T cell compartment alteration/reconstitution [6]. The fact that some B-cell disorders can persist despite successful ART and in absence of apparent disease progression [6–10] suggests that factors other than and/or complementary to viral load and CD4^+^ T cells may contribute to HIV-related B-cell dysregulations. It is unlikely that they result from direct infection of B cells. Indeed, despite the fact that HIV has been shown to replicate in CD40-stimulated B cells *in vitro* [11–13], the virus has not yet been shown to infect or replicate in B cells *in vivo* [6, 11–17]. Moreover, although Epstein-Barr virus (EBV) has been reported to be involved in the AIDS-related B-cell dysregulations leading to lymphomas, only 30–40% of the complications are EBV related and more so the result of chronic stimulation [18].

DCs are involved in the outcome of B-cell development, differentiation and survival, in T-dependent and T-independent manners, mainly through production of the tumour necrosis factor (TNF) family members B lymphocyte stimulator (BLyS/BAFF) and a proliferation-inducing ligand (APRIL) [19, 20]. BLyS is involved in transitional immature (TI) B-cell survival and ontogenesis, and both BLyS and APRIL have been shown to promote B-cell differentiation by inducing molecular events characteristic of class switch recombination (CSR) leading to secretion of isotype switched Ig when in conjunction with signals delivered via the B-cell receptor (BCR) or Toll-like receptors (TLRs) [21–24].

Interestingly, we have recently demonstrated that BLyS overexpressing myeloid DCs (mDCs) are present in the blood of HIV-infected rapid and classic progressors, as soon as in the acute phase of infection and persisting despite successful therapy. Accordingly, these individuals present B-cell dysregulations favouring the overall inflammatory burden and preventing effective viral eradication. In contrast, HIV elite controllers had normal levels of BLyS expression on their blood mDCs, and presented an unaltered blood B-cell compartment. These observations suggest that the extent to which HIV disease progression is controlled may be linked to the integrity of the DC compartment and to its capacity to orchestrate B-cell population dynamics and responses.

## 2. Observations with the HIV Transgenic (HIV-Tg) Mouse System

Early data supporting the hypothesis that DCs are involved in the dysregulation of the B-cell compartment in the context of HIV, were obtained with HIV-Tg mice expressing the coding regions for proteins Rev, Env, and/or Nef of HIV-1, under the control of the human CD4 promoter, and mouse CD4 enhancer, which drive expression in CD4+ T cells, macrophages and DCs [25–27]. HIV-Tg mice develop a disease, which is dependent on *nef* and comparable to many aspects of human AIDS [25, 26]. In these animals, total B-cell numbers were increased in lymph nodes (LN) and spleen, the latter presented a particularly enlarged marginal zone (MZ) [25, 26, 28]. Polyclonal B-cell activation was reflected by hyperglobulinemia and accumulation of anti-dsDNA autoantibodies in serum, as well as by spontaneous hyper-reactivity *in vitro* [28]. The capacity to generate germinal center (GC) reactions and mount matured antibody responses following immunisation was also severely impaired in these animals [28]. Soluble Nef has been shown to penetrate B cells and/or to be propelled via macrophage extensions, suppressing Ig CSR by blocking CD40 signalling and thus impairing the capacity of generating high affinity T-dependent memory B-cell responses [29, 30]. However, B cells enriched from the spleen of HIV-Tg mice behaved similarly to those of littermate controls by CSR in response to anti-CD40 stimulation* in vitro*, suggesting that soluble Nef was not mainly responsible for the impaired capacity of these animals to generate isotype-switched Ig following immunisation [28]. Whether propelling of soluble Nef operates in the context of DC collaboration with B cells remains to be established. The direct effects of Nef on B cells are likely to vary with the status and activation requirements of the different B-cell subpopulations, and DCs may modulate these responses accordingly.

Therefore, as reported in humans, HIV-Tg mice present polyclonal B-cell activation and breakage of tolerance as well as an impaired capacity to generate high affinity adaptive humoral responses. Interestingly, CD11c^+^CD11b^hi^ mDCs from HIV-Tg mice accumulated at entry points of secondary lymphoid organs (SLO) [31], in the LN subcapsular sinus as well as in the MZ of the spleen [28, 32]. Also, mDCs agglomerated among IgM^bright^ plasma cell foci in the red pulp adjacent to the MZ. The fact that blood derived immature mDCs are the primary cells that efficiently capture and transport particulate Ag to the splenic MZ, where they provide signals to Ag-specific MZ B cells [33, 34], suggest that their accumulation at such sites likely contributes to the enlargement of the MZ B-cell population, as well as to the polyclonal activation and breakage of tolerance observed in HIV-Tg animals [28]. This most possibly involves delivery of “altered and/or excessive” contact events and/or B-cell growth factors, such as BLyS, as mDCs from HIV-Tg mice were shown to be altered in their numbers, phenotype and stimulatory functions [32].

BLyS overexpressing mice also present enlarged splenic MZ, B-cell hyperactivity and autoimmunity [35], a phenotype also shared by autoimmune-regulatory-(AIRE)-deficient mice, in which BLyS levels are elevated in serum and overexpressed by peripheral blood CD11c+ DC and stimulated bone marrow-derived DCs [36, 37]. Of note, AIRE is involved in regulation of STAT1 signalling, a pathway shown to be used by the HIV Nef protein to promote a proinflammatory phenotype by human monocyte-derived macrophages [38, 39] and likely in modulation of the over- expression of TNF-*α* by human monocyte-derived DCs [40]. The HIV protein Nef is released early and can be measured in the serum of HIV-infected patients [41]. Furthermore, Nef was reported to penetrate DCs and to alter DC maturation and function, and to induce distinct cytokine/chemokine secretion patterns [1]. Thus early HIV-released products such as Nef may play an important role in modulating DC phenotype, likely influencing the outcome of B-cell disease progression.

Several other HIV products are also likely to influence DCs. Indeed, HIV ssRNA, gp120, and Tat are considered to be major modulators of cellular activation via microbial pattern recognition receptors (PRR), including TLRs, which DC populations express in a wide range, and which prior engagement leads to subversion of the immune response [42]. Interestingly, HIV-ssRNA is recognised by TLR7, which signalling was shown to regulate human monocyte-derived DC-dependent B cell responses through upregulation of BLyS [43]. As such, both mDCs and plasmacytoid (pDCs) enriched from the blood of primary HIV-infected individuals were found to be hyperresponsive to TLR7 agonists and produced high amounts of cytokines and chemokines upon stimulation [44]. HIV-gp120 has also been shown to mediate B-cell polyclonal activation, driving CSR in a BLyS-dependent manner [45]. Altogether, these observations support a role for DCs and BLyS in triggering and driving B-cell dysregulations in the context of HIV.

## 3. HIV Disease Progression: Role for BLyS Overexpressing mDCs in Driving B-Cell Dysregulations

In recent longitudinal studies involving HIV-infected individuals with different rates of disease progression, we have shown that mDC frequencies were reduced in the blood of rapid and classic progressors, as soon as in the acute phase of infection and persisting throughout the course of disease despite successful therapy [46]. The low blood levels of mDCs correlated with increased serum levels of DC-tropic chemokines CCL2, CCL19, and CCL20, suggesting drainage to peripheral sites [47]. Most importantly, our studies have revealed increased levels of BLyS expression in the plasma and on the surface of these blood mDCs [7]. Therefore, mDCs may play a major role in perpetuating B-cell dysregulations, as they overexpress BLyS and are recruited to peripheral sites. Furthermore there might be a pressure towards monocyte-driven differentiation into BLyS overexpressing mDCs, since BLyS overexpressing CD11c^+^CD14^+^CD16^−^ monocytes, precursors of DCs [48, 49], were increased in the blood of chronically infected rapid and classic progressors [7, 46]. Interestingly the murine analogue of the CD11c^+^CD14^+^CD16^−^ population (Gr-1^hi^ monocytes) are linked to the formation of “Tip-DCs” which are a source of inflammatory cytokines and TNF [49].

Consistently, in HIV progressors, B-cell dysregulations were found throughout followup and were accompanied by the increased frequency of a population presenting features shared by both transitional immature (TI) and circulating MZ-like B cells, bearing a CD1c^+^CD21^lo^IgM^hi^CD10^+^CD27^+^ phenotype, which we have termed “precursor/activated MZ-like” B cells [7]. Of course, the human MZ is a complex heterogeneous niche, and MZ-like B cells have been shown to recirculate in humans and are not restricted to the spleen [50]. Further characterization is thus required to identify the exact nature of these B cells. Nevertheless, we believe these cells represent a “first-line” B-cell population that increases in the context of inflammatory conditions such as in HIV infection. These findings are in line with the recently described defects of IgM^+^ “memory” B cells reported for some HIV-infected individuals [6]. TI B cells have been found to be elevated [51] and to preferentially give rise to MZ type B cells in conditions of lymphopenia associated with pathology [52]. The fact that TI B cells are hyperresponsive to BLyS [21] and are increased in the blood of HIV-infected patients with advanced disease [53], suggests that mDC expressing high levels of BLyS may contribute to increased survival of TI B cells and favoured selection into a MZ-like “first-line” B-cell pool [54].

These observations suggest that the DC-mediated B-cell dysregulation process we are proposing would likely, although not solely, affect immature and “first-line” B-cell populations. Given their location in lymphoid organs and mucosal-associated structures, “first-line” B-cell populations are highly influenced by DC and constitute early T cell-independent defence against invading pathogens [22]. Also, given their frequent auto-reactive and cross-reactive repertoires and their relative hyperactivity, these populations are often found in pathologic conditions associated with infections, autoimmunity and lymphomas [21, 22]. This likely involves chronic stimulation and excessive delivery of survival signals [16, 21, 22, 55], which altogether may overcome mechanisms of peripheral tolerance and homeostasis. The aberrant expression of BLyS and/or its receptors is often linked to B-cell autoimmunity and malignancies [21, 56–58]. As such, anergic auto-reactive B cells were shown to evade negative selection when provided with excess BLyS [56, 57]. Recently, excess BLyS was found to be involved in the breakdown of B-cell tolerance in Sjögren's syndrome, favouring expansion of TI and MZ-like B cells [21]. This is reminiscent of events observed during the course of HIV infection, and is in line with correlations between the elevated blood levels of autoreactive antibodies (Abs) and high levels of BLyS expression in the plasma and on the cell surface of blood monocytes in HIV-infected individuals [59, 60].

**Figure 1:**
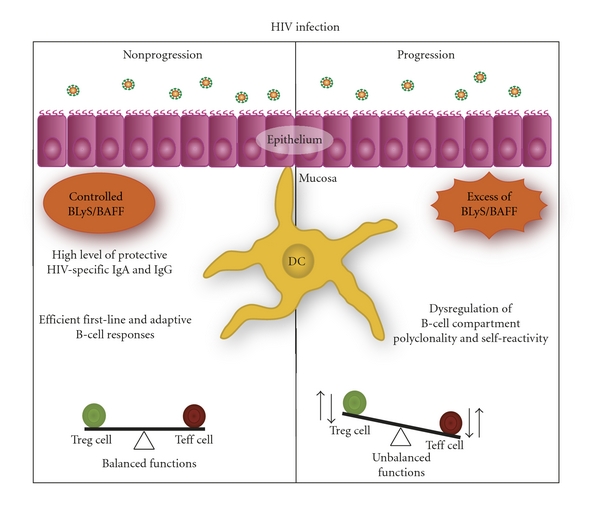
The implication of BLyS/BAFF expression status in the modulation of HIV-specific reponses. Control of HIV infection is reflected by the capacity to regulate BLyS/BAFF expression status at mucosal sites, where the main battle against HIV takes place, promoting efficient HIV-specific B- and T-cell responses, viewed to block systemic invasion by the virus. In contrast, breaching of systemic integrity and progression of HIV infection is reflected by the incapacity to control BLyS/BAFF levels at mucosal sites, leading to dysregulated B- and T-cell responses, impairing the generation of highly protective HIV-specific immunity.

Altogether, the above observations are consistent with the model by which, in the context of HIV disease progression, the high turnover of peripheral DCs may promote the recruitment of BLyS overexpressing monocytic precursors and mDCs, likely contributing to an inflammatory milieu and modulating the outcome of B-cell responses (Figure 1). However, as to whether this process is regulated by the host response and/or modulated by direct and indirect viral effects, remains to be established. Likely explanations involve factors such as HIV-products, excessive apoptosis and release of auto-Ag, as well as products from microbial translocation resulting from breakage of mucosal integrity. Interestingly, levels of products from microbial translocation such as LPS, LBP, and sCD14 were elevated in the blood of HIV progressors harbouring increased frequencies of BLyS overexpressing mDCs and of “precursor/activated MZ-like” B cells [7, 46].

## 4. Control of HIV Disease Progression: Preservation of BLyS Expression Status by mDC Favours Efficient B-Cell Responses

In contrast to that observed in rapid and classic HIV progressors, blood mDC levels and BLyS expression status were unaltered in HIV elite controllers [7, 46]. However, monocytic DC precursors of a CD11c^+^CD14^+^CD16^+^ phenotype, whose murine analogs are thought to settle peripheral organs in steady-state conditions [48, 49], were found to be significantly increased in their blood [46], suggesting high turnover in absence of excessive inflammation. Also, although the blood B-cell compartment remained unaltered in HIV elite controllers, their blood level of mature MZ-like B cells was lower when compared to that in both rapid and classic progressors, as well as to healthy donors [7]. This suggests that recruitment of this “first-line” population to peripheral sites may be beneficial to the host and “control” process. Although our observations in HIV elite controllers may reflect early stages of malfunction, we rather favour the view by which HIV-mediated disease control may be an active process involving DC populations, preventing B-cell dysregulation and favouring the generation of first line as well as effective broadly neutralizing anti-HIV antibody responses. In line with this, the capacity of some HIV-infected individuals to produce potent broadly neutralizing antibodies constitutes a good correlate of prognosis against disease progression [61]. Indeed, the relatively poor antigenicity and silenced immunogenicity of HIV-Env neutralization epitopes preclude the induction of efficient neutralising Abs in most HIV-infected individuals [62].

Needless to say that understanding the generation of such antibodies has become pivotal to the pursue of HIV vaccine research [63].

Given that mucosal DC populations are gate-keepers of peripheral integrity and amongst the first to be involved in the battle against HIV, it is likely that they influence the outcome of mucosal B-cell responses against the virus. IgA is the most abundant mucosal Ig and aids several functions including immune-mediated exclusion of both pathogenic and commensal microorganisms [22, 64]. High levels of mucosal HIV-specific IgA have been found in highly exposed persistently seronegative (HEPS) individuals [65], whereas mucosal HIV-specific IgA responses are rather low in HIV progressors [6]. Although the issue of “protection” conferred by mucosal HIV-specific IgA remains controversial [65], in many studies these Igs have been found to neutralize HIV infection and inhibit viral transcytosis *in vitro*. A recent study on HIV-gp41-specific mucosal IgA, produced by cervical B cells from HEPS individuals, demonstrated evidence for hypermutation and affinity maturation [66]. These observations based on natural control/immunity versus HIV suggest that efforts to develop an effective vaccine against HIV should consider soliciting HIV-specific mucosal IgA production. In support of this are the recent findings by Bomsel et al. demonstrating that mucosal IgA and IgG, elicited through mucosal vaccination with HIV-1 gp41 subunit virosomes in nonhuman primates, prevented systemic invasion following vaginal simian-HIV challenge, by blocking transcytosis and by mediating antibody-dependent cellular cytotoxicity (ADCC) [67]. Importantly, these animals lacked serum neutralizing antibody activity, highlighting the role of effector antibodies at the mucosal portal of entry, and their importance in preventing dissemination of HIV infection [68]. In humans, the Thai RV144 vaccine trial has been raising some hope. Although the nature of the immune responses responsible for the modest protection conferred (31%) have yet to be unravelled, it appears that the RV144 vaccine regimen may have elicited transient protective B-cell responses, which nature in terms of generation and effector mechanisms has become critical to define, and appears to involve short-lived antibody responses viewed to block HIV transmission at mucosal surfaces [69]. However, in light of the protection correlates data released at the AIDS Vaccine 2011 meeting in Bangkok, (J. Kim, B. F. Haynes and colleagues), high levels of plasma Env IgA, most probably monomeric, correlated with a 54% increased risk of infection. This is in line with our findings showing that rapid and classic HIV progressors presented hyper-IgA in their serum when compared to slow progressors and elite controllers [7]. Moreover, the fact that BLyS levels were increased in the blood and on the surface of mDC in rapid and classic progressors may have favoured serum hyper-IgA production. Indeed, overexpression of BLyS in Tg mice has been shown to favour monomeric hyper-IgA CSR by spleen MZ B cells [70], a population known to present enhanced IgA CSR potential [71], and which we have shown to be activated and increased in the blood of rapid and classic HIV progressors [7]. Nevertheless, production of serum and mucosal IgA appear to follow different circuits, and requirements may differ depending on the sites of induction, the immunomodulatory milieu, and B-cell populations responding [72]. It is, therefore, possible that high levels of monomeric IgA in the blood may increase risk of infection and systemic invasion, whereas the production of dimeric IgA at mucosal sites may confer protection. Unfortunately, there were no mucosal samples in the RV144 trial to assess mucosal dimeric Env IgA levels, which we would predict may constitute better correlates of protection.

Again, based on these observations, our model would suggest that systemic and mucosal BLyS expression status, likely contributes to the modulation of B-cell responses against HIV. On one hand, BLyS expression patterns at mucosal ports of entry may promote mucosal IgA and also IgG, viewed to block systemic invasion by the virus. On the other hand, the incapacity to control these levels and constrain this immune activity to mucosal sites may allow breaching systemic integrity and perpetuating disease (Figure 1).

## 5. The Importance of DCs at Mucosal Sites

DCs are involved in maintaining a balance between tolerance versus protective immunity at both the innate and adaptive levels, which process is pivotal at mucosal sites, where immune homeostasis processes warrant peripheral integrity, and where the main battle with HIV takes place [1–4]. Recent studies have demonstrated the importance of cross-talk between epithelial cells and mDC populations in maintenance of a homeostatic balance of regulatory versus inflammatory responses at the mucosal level [73–75]. In the murine gut, two mDC populations have recently been given importance, the CD103^+^ and CD103^−^ mDC populations which derive from distinct precursors and are found in the mucosal associated lamina propria and draining lymphoid organs [76, 77]. Murine gut lamina propria CD103^+^ mDC are known to support the generation and retention of IgA-producing B cells in the lamina propria through retinoic acid (RA) production [78]. Also, the tolerogenic capacity to modulate CD4^+^ T regulatory (Treg) lymphocytes is conferred by CD103^+^ mDC through transforming growth factor (TGF)-*β* and RA-dependent mechanisms [73]. On the other hand, the CD103^−^ mDC population was shown to produce higher levels of proinflammatory cytokines and generate protective immunity [48], promoting the differentiation of CD4^+^ T mucosal effector lymphocytes [79]. Creating imbalance in these mDC populations by favouring CD103^−^ mDC reconstitution leads to experimental-induced colitis in a TNF-*α*-dependent manner [76, 77]. Analogous mucosal mDC populations have been described in the human gut [73] and more recently in the human lung, where they have been shown to differentiate from monocyte populations [80]. Based on these observations, it is tempting to speculate that in the context of HIV infection, the incapacity to keep a balance in homeostatic mucosal mDC populations, may allow for increased “proinflammatory” mDC, possibly overexpressing BLyS, to accumulate at mucosal sites, where they contribute to the imbalance of T regulatory/effector ratios and modulate the outcome of mucosal B-cell responses.

Although IgA^+^ plasma cells are generated mainly in the mucosal-associated lymphoid tissue (MALT) through a T-dependent mechanism, IgA^+^ cell differentiation has also been shown to be modulated in the MALT in a T-independent fashion through factors such as RA and cytokines such as BLyS and APRIL produced by mDCs. Also, mDCs were found to support T-independent IgA class switch recombination in human lamina propria via production of APRIL [22, 64]. Interestingly in mice, both conventional B2 and first-line IgM^+^ peritoneal B1 cells, have been shown to migrate directly to the gut lamina propria and differentiate into IgA producing cells [64], through help provided by lamina propria mDCs. Importantly, TLR-mediated epithelial cell:DC cross-talk at the level of human tonsillar crypts was shown to orchestrate B-cell CSR through modulation of BLyS levels via epithelial cell secretion of thymic stromal lymphopoietin (TSLP) [75]. Work by Fontenot et al. [81] demonstrated that HIV induces human genital mucosal epithelial cells to produce TSLP, activating mDCs, which in turn promote HIV-1 replication in CD4^+^T cells. Furthermore, in rhesus macaques, increased TSLP expression was found to be concurrent with viral replication in the vaginal tissues within the first 2 weeks after vaginal SIV exposure. Therefore, these studies suggest that levels of TSLP involved in the cross-talk between mucosal epithelial cells and mDCs may contribute to modulating BLyS levels, and this may be important in modulating the fate of HIV infection in mucosa, and the outcome of disease progression.

Based on these observations, it is reasonable to think that the incapacity to keep a balance in homeostatic epithelial cell:DC cross-talk processes, which is likely to occur in individuals who progress with HIV infection, will promote inflammation and lead to disease perpetuation. In contrast, the capacity to maintain immune homeostasis at mucosal sites may allow for better control of immune responsiveness and HIV infection. This view is consistent with a report showing that early prevention of macrophage inhibitory protein (MIP)-3*α* (CCL20) production in the genital tract of SIV-susceptible female macaques prevented excessive recruitment of pDC and mDC populations, establishment of an inflammatory milieu and infection, despite repeated intravaginal exposure to high doses of SIV [82]. Furthermore, studies of SIV infection in nonpathogenic animal models have shown that their control of disease progression appears linked to better management of the aberrant immune activation by early onset of anti-inflammatory IL-10 production and T regulatory activity. Mucosal Th17 effectors are the main targets for HIV/SIV in the gut and massive depletion of these cells [83–86] contributes to the breakage of integrity of the mucosal barrier and microbial translocation from the gut, characteristic of pathogenic infections [87]. Fewer, Th17 effector target cells were generated in nonpathogenic than in pathogenic SIV infections [88], a process linked to a low type I interferon- (IFN-) gene profile and low TLR7-signalling [89]. Interestingly, both type I IFN- and TLR7-signalling have been shown to be involved in regulation of BLyS expression patterns by DC populations [43, 90].

## 6. Role of DC in Modulating B-Cell Effector/Regulatory Responses in the Context of HIV

The influence of DC in the outcome of B-cell responses against HIV may modulate the outcome of CD4^+^ T-cell effectors/targets for the virus. Indeed, there is an increasing body of experimental evidence demonstrating the role of B cells in regulating the development, proliferation and maintenance of CD4^+^ effector, memory as well as regulatory T cell populations, through both contact and/or cytokine mediated effector/regulatory functions [91, 92]. The increased lymphotoxin “effector” to IL-10 “regulatory” B-cell ratio was recently demonstrated in the pathophysiology of multiple sclerosis (MS), promoting proinflammatory T cell effector functions [93]. MS patients treated with a single course of Rituximab, a monoclonal antibody that selectively targets and depletes CD20^+^ B lymphocytes but not plasmablasts, presented lower inflammatory brain lesions and clinical relapses, characterised by the reduction in effector T lymphocyte infiltrates as well as decreased proinflammatory Th1 and Th17 responses [94].

Episodic depletion of B cells is an effective therapy for several T cell-mediated autoimmune diseases by promoting the emergence of regulatory B-cell populations that will hopefully prevent the reactivation of remaining autoreactive T cells [91]. The fact that at low concentrations BLyS was shown to favour IL-10-production by murine splenic MZ “regulatory” B cells, whereas elevated BLyS concentrations rather promoted MZ B-cell activation and differentiation, suggests that BLyS may play an important role in modulating the nature of B-cell functions and subsequently the outcome of Treg/T effector balance [95]. As such, blocking of BLyS, which has been used as a therapeutic target in clinical trials for the treatment of autoimmune disorders such as systemic lupus erythematosus (SLE) [58], may also be efficient in modulating this balance. In this view, decreased effector CD4^+^ T cell functions and increased regulatory CD4^+^ T cell functions were observed following treatment of NOD mice (model for type I diabetes) with the B-cell maturation antigen (BCMA)-Fc construct, which blocks BLyS-mediated survival signals for B cells. In a collagen-induced model of rheumatoid arthritis, BLyS overexpression was shown to promote the expansion of Th17 effector cells, and BLyS gene silencing inhibited DC driving of Th17-cell differentiation *in vitro* [96]. These observations suggest that DCs may influence T cell differentiation and effector target CD4^+^ T cell availability for HIV in a BLyS-mediated manner either directly and/or indirectly via modulation of B-cell functions.

## 7. Concluding Remarks

The extent to which HIV disease progression is controlled may be linked to the integrity of the DC compartment and BLyS expression status, and to its capacity of orchestrating B-cell population dynamics and responses. This may be best achieved at mucosal sites, where DC populations present an inherent capacity of modulating the balance between tolerance and protection, and are amongst the earliest cell types to be exposed to the virus. It is, therefore, likely that they influence mucosal B-cell responses against HIV, which in turn modulate the outcome of CD4^+^ T cell effectors, prime targets for the virus. The early assessment of BLyS levels as well as DC and B-cell population statuses have great prognostic value in predicting the clinical course of HIV infection. This should be borne in mind in the design of future preventive vaccines, which should aim at inducing first line as well as adaptive mucosal B-cell responses to block systemic invasion by the virus at the initial site of exposure. Therapeutic approaches viewed to control BLyS levels may also be promising to reduce both HIV target cells and systemic immune activation that are the hallmarks of HIV disease progression and AIDS-associated diseases such as cancers, autoimmune, and cardiovascular disorders.

##  Funding

This work was supported by grants from the Canadian Institutes of Health Research (CIHR) and the Réseau SIDA from the Fonds de la Recherche en Santé du Québec (FRSQ). M. Roger is recipient of a Research Scholar award from the FRSQ.
